# Ion-Exchange Membrane-Centric Durability Testing and Degradation Characterization for Industry-Relevant CO_2_ Reduction

**DOI:** 10.1021/acs.iecr.5c02103

**Published:** 2025-10-16

**Authors:** Christopher D. Sewell, Danielle A. Henckel, Michael G. Resch

**Affiliations:** 53405 National Renewable Energy Laboratory, Golden, Colorado 80401, United States

## Abstract

Electrochemical CO_2_ reduction is a promising conversion process for producing value-added fuels and chemicals from electricity and CO_2_ as a sustainable carbon feedstock to domestically produce fuels and chemicals from industrial waste. Having reached industrially viable performance metrics with small-scale CO_2_ electrolysis cells, the field must now increasingly focus on extending the device durability of large stacks to achieve equivalent metrics for 35,000+ hours to decrease maintenance and capital costs. Reported device lifetimes have increased in recent years, with the longest stability studies for CO, ethylene, and formic acid production being published in 2024–2025 with operation times of 4500, 1000, and 5200 h, respectively. Unfortunately, significant extension of the device durability is still required. Here, we provide an overview of ion-exchange membranes (IEMs) and provide insight into the variety of degradation mechanisms that must be overcome to enable the community to meet durability targets. In an effort to accelerate the extension of device lifetimes, we propose a general approach for characterizing CO_2_ electrolysis cell degradation before and after durability testing to better elucidate the mechanisms and failure modes of IEMs in zero-gap cells. Furthermore, we encourage the adoption of operando characterizations in tandem with accelerated stress and durability tests, postulating that their combined applications will be increasingly valuable. We hope that this perspective motivates future durability studies to evaluate degradation across the entire electrolysis cell.

## Graphical abstract

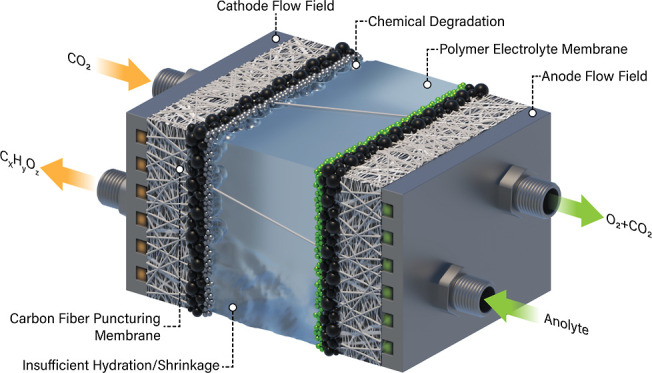

## 1 Introduction

Electrochemical CO_2_ reduction (CO_2_R), also referred to as CO_2_ electrolysis (CO_2_E), is viewed as a promising technology to convert concentrated industrial CO_2_ emissions into value-added fuels and chemical feedstocks. CO_2_R can strengthen supply chains, reduce reliance on imported commodities, and expand the production of locally sourced chemical products.

To reach industrial viability for CO_2_R electrolyzer technology, technoeconomic analysis (TEA) has shown a consistently high product formation rate (current density ≥ 200 mA/cm^2^), a high product selectivity/faradaic efficiency (FE > 80%), and high energy efficiency (EE) (reaction potential ≤ 3 V) are required.^1^ As a result of extensive research on CO_2_R technology, these performance metrics have been achieved at laboratory scales (5–250 cm^2^ cells), which is in no small part due to the advancement of CO_2_R cell configurations and intervention strategies. Early studies of CO_2_R were performed in H-cell configurations, named after the typically H-shaped glass cell utilized, which comprise a cathode and an anode chamber separated by an ion-exchange membrane (IEM) (Figure 1a).^2^ Typically, the cathode chamber contains a working electrode and a reference electrode suspended in an electrolyte, termed a catholyte to reference its location in the cathode compartment, that is continuously pumped with CO_2_, while the anode chamber holds the counter electrode suspended in another electrolyte, termed an anolyte to reference its location in the anode compartment. While H-cells prove useful in evaluating the performance of various electrocatalysts and electrolytes due to their simple design, they are unable to attain high current densities because of limited CO_2_ solubility and mass transfer within electrolytes.^3^ To overcome these deficiencies, gas diffusion electrode (GDE)-based cells were developed. A GDE commonly takes the form of a square-porous electrode consisting of three layers, a hydrophobic macroporous carbon fiber layer, a hydrophobic microporous carbon fiber layer, and a catalyst/binder layer, allowing gas to flow through the back of the electrode and react at a gas|catalyst|electrolyte triple-phase interface.^4^ Initially, these GDEs were utilized in hybrid cells that consisted of an anode GDE|IEM|catholyte|cathode GDE configuration (Figure 1c). Anolyte is introduced into the hybrid cell via the back of the anode GDE to reach the catalyst|IEM interface, while CO_2_ flowed into the cell through the back of the cathode GDE, reaching the catholyte|catalyst interface where CO_2_R occurs. By removing the need to saturate the catholyte with CO_2_, these hybrid cells greatly increased the rate of transfer of CO_2_ to reaction sites. While hybrid cells represented a significant improvement over H-cell technology, they suffer from higher operating voltages resulting from the catholyte and electrolyte flooding of the cathode GDE when scaled up for higher current densities.^3^

**Figure 1:**
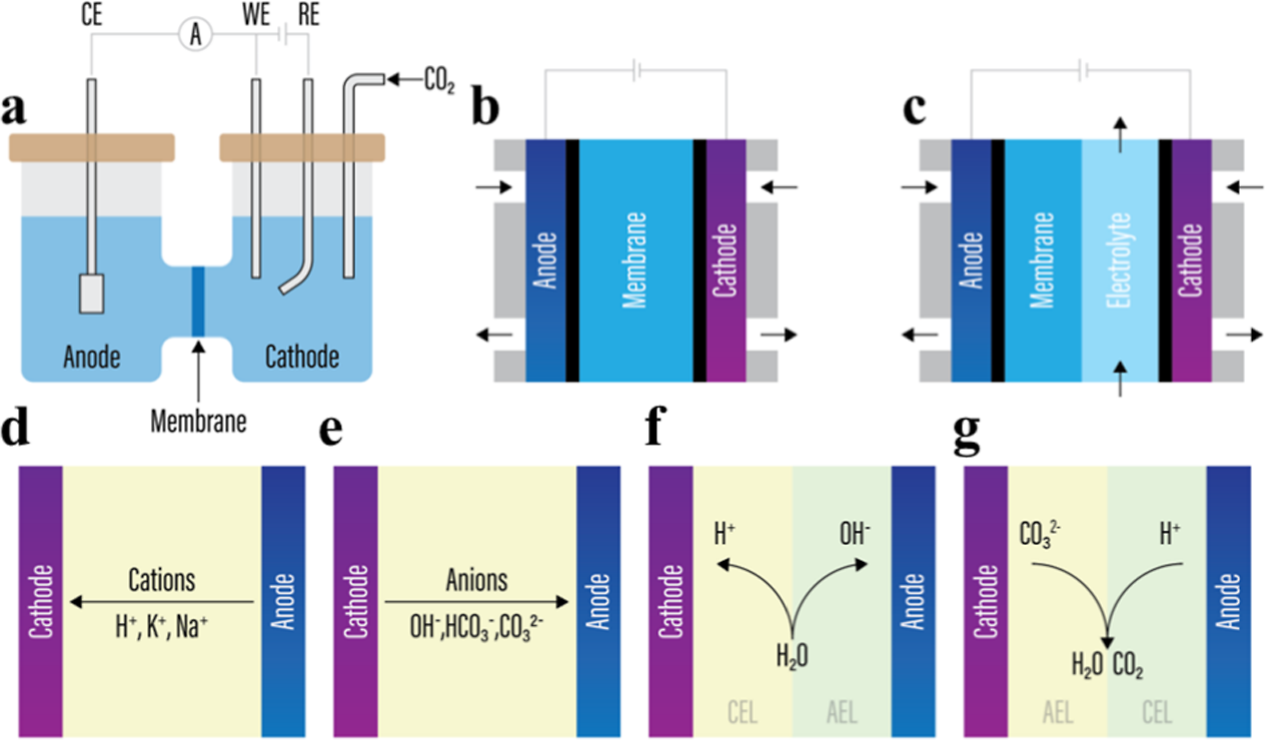
Illustration of (a) an H-cell configuration, (b) a zero-gap cell configuration, (c) a hybrid cell configuration, and (d–g) several commonly used IEMs in zero-gap configurations. Namely, (d) a CEM in which cations travel from the anode to the cathode, (e) an AEM in which anions travel from the cathode to the anode, (f) a reverse-bias BPM in which water dissociates at catalysts sandwiched between a CEL and an AEL, and (g) a forward-bias BPM in which water and CO_2_ are formed between an AEL and a CEL.

Drawing inspiration from commercial fuel cells, hybrid cells were further improved upon by utilizing membrane electrode assemblies (MEAs), in which both the anode and cathode GDEs are in direct contact with the IEM. This cell is termed an MEA-based cell, or a zero-gap cell, referencing the absence of the electrolyte between the electrodes and IEM (Figure 1b). Typically, CO_2_ is humidified prior to flowing into the cathode GDE, and the anolyte is similarly transported to the anode catalyst layer through the back of the anode GDE. By removing the intermediating electrolyte layer, zero-gap cells provide decreased ion transport resistance, increased CO_2_R reaction rates, decreased chances of GDE flooding, and increased system EE.^3^ While the adoption of zero-gap cells has led to higher CO_2_R device performance, significant advancements in electrolyzer durability are still needed to reach industrial viability, with a recent TEA modeling study by Grim et al. reporting required minimum device lifetimes of >4 years (>35,000 h).^1^

Identifying an accurate average CO_2_ electrolyzer lifetime is difficult due to the variation of catalyst/electrodes, IEMSs, cell configurations, and targeted products reported in the literature. To assist in clarifying the current state of the CO_2_R device durability, publications from the last 8 years demonstrating extended electrolyzer runs employing zero-gap or hybrid cell configurations were collected and reviewed. The reported device lifetimes and performance metrics are compiled into Table 1. In most of the durability studies found, electrochemical testing was halted after ∼100–200 h (Figures 2a/1b).^5–16^ Nevertheless, there has been an emerging number of extended durability studies in recent years, with 3/5 studies detailing device lifetimes ≥1000 h having been published in 2024–2025 (Figure 2c).^16–20^ The compiled data also suggests some recent success in extending CO_2_ electrolyzer durability, with the longest stability studies for CO, ethylene, and formic acid production being published in 2024–2025 with operation times of 4500, 1000, and 5200 h, respectively (Figure 2d).^16,19,20^ Despite recent progress in extending electrolyzer longevity, the gap between current cell durability and the economically required device lifetimes remains vast.

**Table 1:** Compilation of Reported Lifetimes and CO_2_R Performance Metrics from Durability Studies Published from 2017 to 2025^a^

IEM	active area (cm^2^)	*E* (V)	*i* (mA/cm^2^)	product	FE (%)	durability	year	refs
AEM-Sustainion	2	3.2	100	CO	90	100	2022	^5^
AEM-Sustainion	2	3.57	75	CO	83	103	2022	^6^
AEM-Sustainion	2.25	2.6	100	CO	45	200	2025	^7^
AEM-Sustainion	5	3	200	CO	90	1000	2017	^17^
AEM-Sustainion	5	3	200	CO	98	3800	2018	^18^
AEM-PiperION	5	3	180	CO	80	100	2025	^8^
AEM-PiperION	8	3.2	420	CO	90	200	2021	^9^
AEM-PiperION	4	3.3	100	CO	90	1000	2025	^19^
AEM-PiperION	1	3.3	100	CO	80	2000	2025	^19^
AEM-PiperION	100	3.5	100	CO	80	2000	2025	^19^
AEM-PiperION	100	3.5	100	CO	80	4500	2025	^19^
AEM-QAPPT	3.2	2.25	100	CO	90	100	2019	^10^
AEM-Aemion	5	3.27	300	CO	90	100	2023	^11^
AEM-PVDF porous	100	3	200	CO	80	110	2024	^12^
CEM-Nafion	5	4.3	60	CO	99	50	2022	^23^
BPM-Fumasep	5	3.25	100	CO	90	100	2025	^13^
BPM-Fumasep	4	3.6	200	CO	90	350	2025	^24^
AEM-Sustainion	800	4	100	CO/C2H4	20/20	240	2024	^14^
AEM-Sustainion	5	3.65	600	C_2_H_4_	64	190	2019	^15^
AEM-Sustainion	5	3.65	230	C_2_H_4_	64	190	2019	^15^
BPM-Fumasep/Nafion	30	4.4	330	C_2_H_4_	50	1000	2024	^16^
CEM-Nafion	5	3.6	100	HCOOH	90	60	2023	^25^
CEM-Nafion	1	2.2	600	HCOOH	91	5200	2024	^20^
BPM-Sustainion/Nafion	5	3.4	140	HCOOH	31	550	2017	^26^

a Identified studies utilized zero-gap, hybrid cell, or cell stack configurations, while employing a variety of IEMs and targeting the products CO, C_2_H_4_, or HCOOH. QAPPT—quaternary ammonia poly­(*N*-methyl-piperidine-*co*-*p*-terphenyl). PVDF—polyvinylidene fluoride.

**Figure 2:**
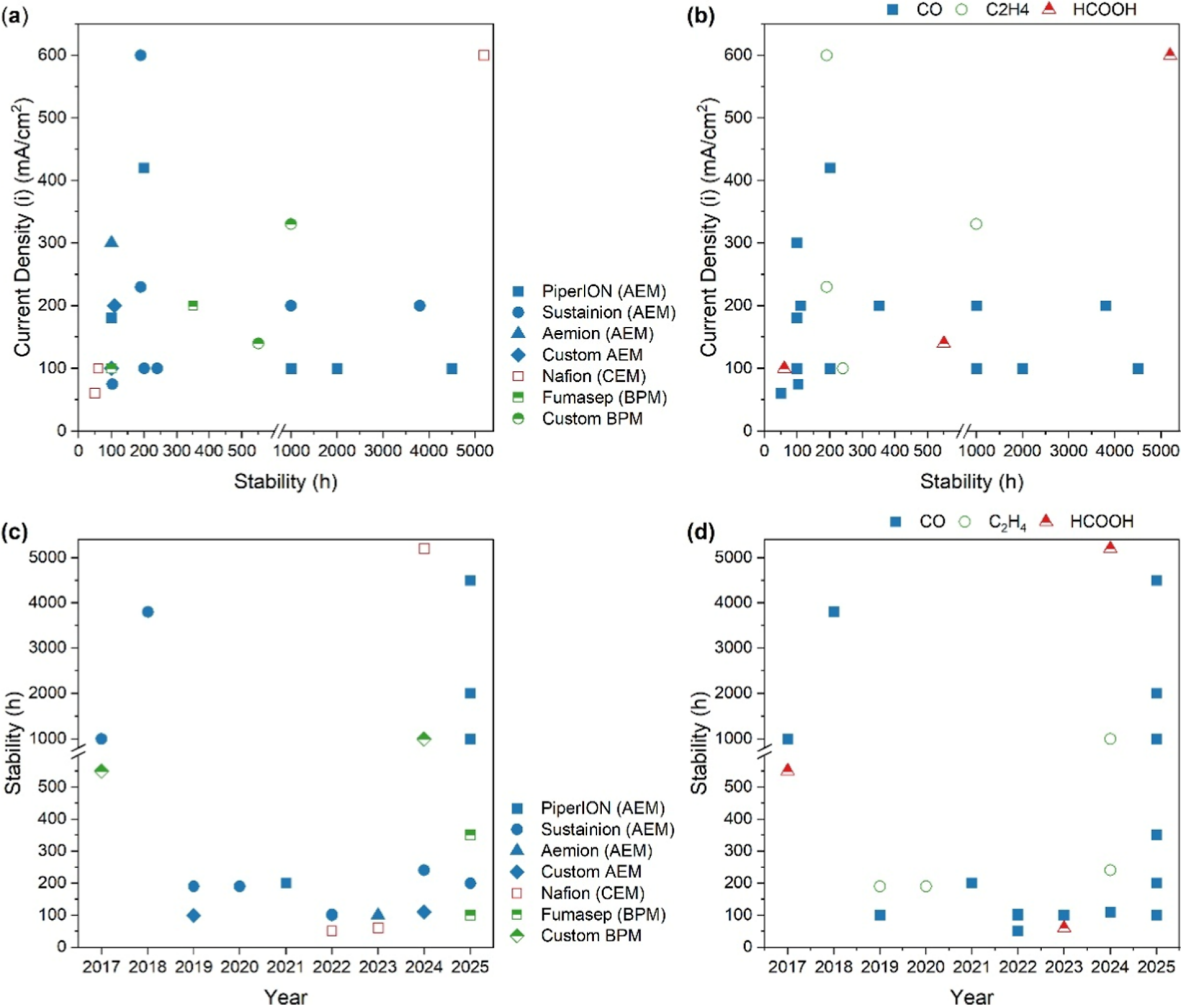
Visualization of reported CO_2_ electrolyzer lifetimes from various works published from 2017 to 2025. The comparison of reported device lifetimes and current densities is separated by (a) the IEM employed by each study and (b) the targeted products. Additionally, the published electrolyzer stability is displayed with reference to the year of the study, separated by (c) the IEM employed by each work and (d) the targeted products.

To address this gap, it is paramount to develop a more encompassing understanding of degradation mechanisms and failure modes within CO_2_ electrolyzers. While several researchers have shifted their focus to improving CO_2_ electrolyzer durability, most published works have focused solely on improving GDEs and catalyst lifetimes.^4^ IEMs control ion transport between the cathode and anode, influence the localized pH at both electrodes (especially in zero-gap configurations), and act as separators, preventing electrical shorting and product crossover; as IEMs heavily influence both the FE and operating voltage (EE) of CO_2_R devices, increased efforts need to be directed toward the characterization of IEM degradation mechanisms within MEA-based devices to accelerate the improvement of electrolyzer lifetimes.^21,22^ The limited attention given to IEM and MEA degradation likely stems from the lack of well-defined durability testing standards for these components. Currently, no widely adopted industrial practices exist for CO_2_ electrolysis durability testing, particularly for IEM evaluation, underscoring the need for standardized methodologies as the technology moves toward commercial deployment.

The goal of this perspective is to identify research opportunities in CO_2_ electrolyzer durability at industrially relevant currents, with an added focus on elucidating the degradation mechanisms of IEMs. Herein, we present a brief overview of the three main IEMs utilized in CO_2_ electrolysis, as well as the commonly employed cell configurations in the literature. Next, an overview of the primary IEM degradation mechanisms and their effect on device performance is given. We do not provide an in-depth review of the current literature in these areas in order to keep this perspective concise and instead refer readers to the following detailed reviews,^3,4,21,22,27,28^ which provide necessary background information and cover recent advancements in IEMs,^21,22,27^ CO_2_ electrolyzer scale-up studies,^3^ GDE/catalyst degradation mechanisms,^4^ and operando methodologies.^28^ Finally, we propose a suite of techniques for CO_2_ electrolyzer durability testing and the investigation of MEA-based cell degradation mechanisms, including procedures specific to the study of IEM degradation and outlining the future prospects of operando characterizations in combination with accelerated testing methods.

## 2 Background: A Brief Overview of IEMs in CO_2_ Electrolyzers

There are three types of IEM that are commonly utilized in CO_2_ electrolyzers: anion-exchange membranes (AEMs), cation-exchange membranes (CEMs), and bipolar membranes (BPMs). AEMs (Figure 1e) are designed to selectively allow the transport of anions, ideally OH^–^, across the cell by incorporating cationic functional groups into their structures and, to date, have demonstrated the best CO_2_-to-CO electrolysis performance in terms of FE and EE.^22,29^ This superior performance stems from the high pH environment that forms at the cathode|AEM interface caused by the continued production of OH^–^ during CO_2_R that helps shift reaction selectivity away from the competing hydrogen evolution reaction (HER).^30^ Unfortunately, the application of AEMs still faces significant challenges, such as their susceptibility to nucleophilic OH^–^ attacks on their immobilized functional groups or polymer backbone that result in an innate instability in alkaline environments like KOH or NaOH electrolytes (detailed in Section 3.1).^21^ Moreover, CO_2_ rapidly reacts with OH^–^ to form carbonate anions, which then transfer across the AEM to the anode chamber. Not only does this decrease the ion-transfer rate across the membrane, as the bulkier HCO_3_^–^ and CO_3_^2–^ ions become the primary charge carriers, but it also creates a CO_2_ pumping effect where the carbonate anions are oxidized at the anode to release CO_2_. Additionally, rapid cell failure can occur due to a buildup of carbonate ions at the cathode that results in carbonate salt precipitation.^21,22,30^

In comparison to AEMs, CEMs (Figure 1d) are relatively well developed from their extensive use in proton-exchange membrane (PEM) fuel cells and PEM water electrolyzers, lending them to enhanced chemical stability over AEMs in CO_2_R applications.^21,27^ However, fuel cell studies have shown hydrogen peroxide (H_2_O_2_) causes aggressive chemical degradation in PEMs.^31^ CEMs are designed to selectively allow the transport of cations and protons across the cell by incorporating anion functional groups into their structures. CEMs create a low pH environment at the cathode|CEM interface, shifting reaction selectivity toward HER; as a result, CEMs are more commonly utilized in hybrid cells (Figure 1c), and H-cells (Figure 1a), where an electrolyte buffer layer exists between the cathode and CEM.^22^ Despite yielding lower CO_2_R performance in terms of FE and EE, CEMs suppress the carbonate formation phenomenon, thereby effectively eliminating concerns of CO_2_ pumping and carbonate salt precipitation.^32^

The third type of IEM utilized in CO_2_ electrolyzers, BPMs, comprises an anion-exchange layer (AEL) and a cation-exchange layer (CEL) laminated together. BPMs can be used in two different modes, referred to as forward bias (Figure 1g), where the AEL is in contact with the cathode, and reverse bias (Figure 1f), where the CEL is in contact with the cathode.^22^ During BPM operation in the forward bias mode, H^+^ and OH^–^ counterions combine at the AEL|CEL interface to form H_2_O, providing the advantage of a high localized pH at the cathode|BPM interface and the prevention of carbonate anion crossover into the anode chamber by the CEL, mitigating CO_2_ losses at the anode. Unfortunately, the generation of species like CO_2_ or H_2_O from counterion combination at the AEL|CEL interface can result in the delamination of the BPM if there is insufficient porosity between the two layers.^21,33^ One strategy employed by Hu et al. to overcome this deficiency was a forward bias BPM with a perforated CEM (perforated cation-exchange membrane (PCEM)), which allowed formic acid and other species present at the BPM interface to vacate the cell through the anode GDE and flow field (Section 3.2).^34^

Alternatively, BPM operation in the reverse bias mode, in which H_2_O is dissociated at the CEL|AEL interface with the aid of catalysts, provides the advantage of mitigated ion crossover, thereby allowing the use of a different catholyte and anolyte (when employing hybrid cells). Reverse bias BPM configurations have been demonstrated to inhibit product crossover for both neutral and anionic species due to the outward fluxes of OH^–^ and H^+^ generated by water dissociation at the CEL|AEL interface.^35^ Moreover, the reverse bias mode mitigates salt precipitation on the GDEs, providing stable product formation.^21,22^ While promising, reverse bias BPMs create a low localized pH at the cathode|BPM interface, increasing the selectivity of the competing HER. Reverse bias BPMs also suffer from increased operating potentials because of the water dissociation at the membrane junction.

## 3 IEM Degradation Mechanisms

The routes through which membranes degrade can be split into two categories, chemical degradation and mechanical degradation, which are defined as any unintended/undesired changes that occur through a chemical or mechanical process, respectively. Chemical degradation typically occurs via chemical attacks on the IEM polymer backbones or ionic functional groups, resulting in membrane thinning, reduced mechanical integrity, and decreased ionic conductivity. Traditional AEMs such as quaternary ammonium (QA)-functionalized poly­(arylene ethers) are considered to have poor chemical stability under high pH conditions and degrade primarily by direct nucleophilic OH^–^ substitution and Hofmann elimination.^21,29–31,36,37^ On the other hand, CEMs are considered chemically stable, lasting >60,000 h in water electrolyzers. While the chemical degradation of CEMs is not well documented in CO_2_R, water electrolysis and PEM fuel cell studies have shown that hydrogen peroxide (H_2_O_2_), hydroxyl radicals (HO^•^), and peroxyl radicals (HO^•^_2_) will readily attack the membrane via chain scission.^21,31,36,38,39^

Mechanical degradation most often occurs during operation due to membrane expansion and a resulting increase in mechanical strain in the cell or during membrane preparation and cell assembly and compression, where pinholes, cracks, or tears form on the IEM, significantly reducing device lifetimes. Additionally, BPMs can undergo delamination of the AEL and CEL during operation, drastically decreasing cell performance.^21^ In the following sections, each of the described IEM degradation mechanisms will be discussed in more detail.

### 3.1 Chemical Degradation

Chemical degradation within IEMs primarily occurs through two general pathways, a chemical attack on the immobilized ion-exchange functional groups or the polymer backbone via direct nucleophilic OH^–^ substitution (Figure 3b,d) and Hoffmann elimination (Figure 3c).^21,29–31,36,37^ Attacks on the ion-exchange functional groups reduce the IEM ionic conductivity, increasing the ohmic resistance in the cell. This results in an increased operating overpotential and decreased EE.^27^ On the other hand, chemical attacks on the polymer backbone are less straightforward; deterioration of the IEM polymer backbone typically results in membrane thinning, which can initially have a beneficial effect on cell performance.^40^ Reducing the IEM thickness decreases the through-plane ohmic resistance, leading to a decreased operating overpotential and increased EE. However, excessive membrane thinning will reduce IEM mechanical integrity and initiate the formation of pinholes, greatly increasing the gas crossover rate. Pinhole formation can quickly reduce both the FE and EE of an electrolyzer and possibly cause an electrical short.^41^

**Figure 3:**
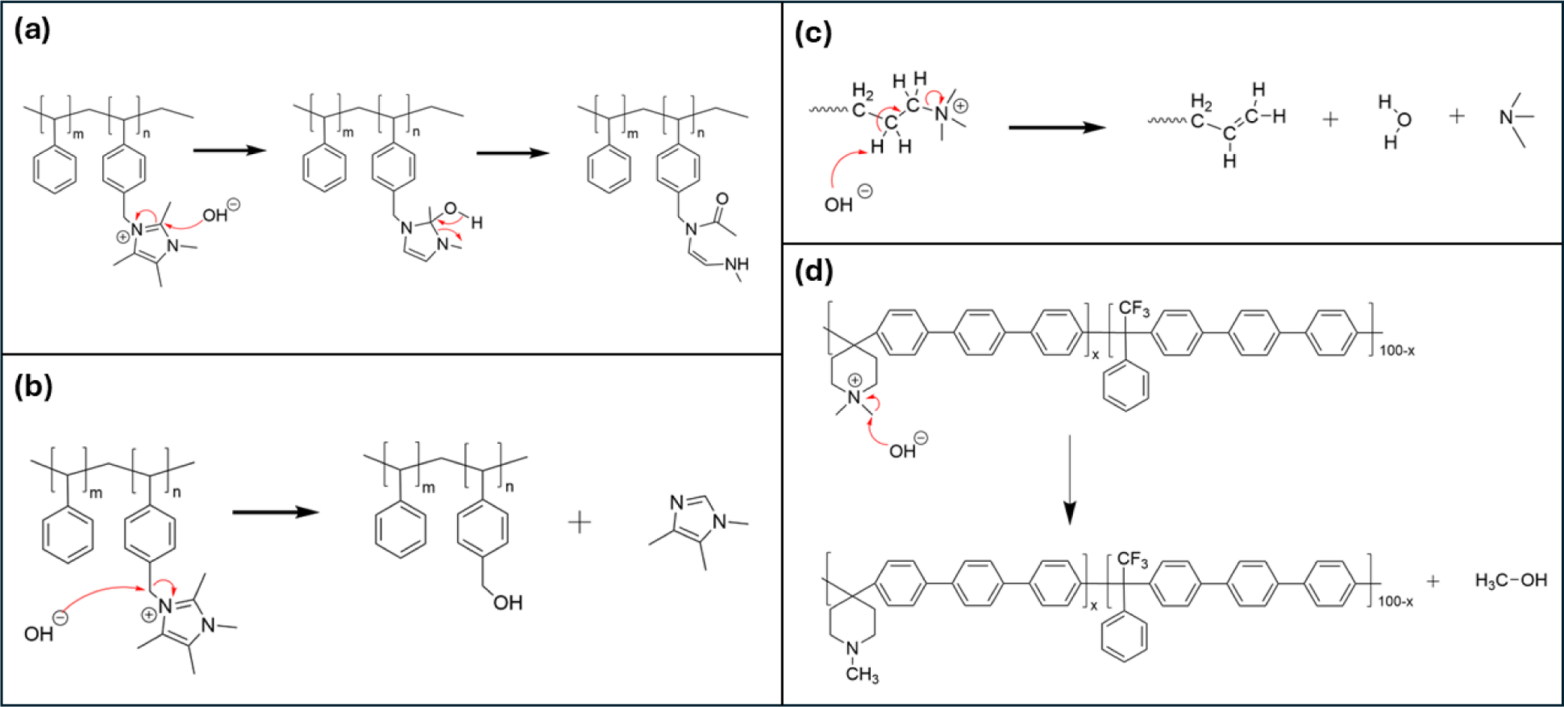
Common chemical degradation pathways of the AEMs. Specifically shown is the degradation of (a) Sustainion via imidazolium ring opening, (b) Sustainion via nucleophilic substitution (S_N_2) at benzylic carbon, (c) QA via Hoffman elimination, and (d) PiperION via S_N_2 at methyl. These chemical degradation pathways are not specific to any AEM.

Recent work in the context of AEM water electrolyzers has shown that ionomer backbone degradation can be triggered by ring-opening reactions of benzimidazolium or imidazolium groups (Figure 3a), especially under oxidative conditions such as those near the anode.^42^ These degradation pathways are likely to also occur in CO_2_ electrolyzers operating under alkaline conditions given the shared chemical environment and membrane chemistries.

In AEM-based electrolyzers, chemical degradation is primarily caused by nucleophilic OH^–^ attacks; this is worsened by the nature of AEM functionality, which creates an alkaline environment at the cathode|AEM interface.^30^ Nucleophilic OH^–^ attacks on AEMs are exacerbated when water content is low due to the continuous consumption and production of H_2_O and OH^–^, respectively, at the cathode resulting in high pH and weakly hydrate OH^–^ that is aggressively nucleophilic.^27^ Thus, it is imperative that the membrane, especially in a zero-gap cell, receives adequate hydration via a humidified CO_2_ gas stream and electro-osmotic drag from the anolyte. In contrast, AEMs in H-cells and hybrid cells receive ample hydration from the presence of both the anolyte and catholyte.

CEMs boast significantly higher chemical stability than AEMs, so there is a deficit of information on their degradation during CO_2_R.^21^ In water electrolysis, reactions between O_2_ and H_2_ on the cathode can generate small concentrations of H_2_O_2_ and other reactive species such as hydroxyl (OH^•^) and peroxyl (HO_2_^•^) radicals. These radicals attack the CEM via chain scission of the typical perfluorosulfonic acid (PFSA) polymer backbone, reducing polymer molecular weight and membrane IEC, conductivity, and thickness.^31,39^ AELs in BPMs are susceptible to the same chemical degradation mechanisms as AEMs, which are of more concern than the slow degradation of CEMs/CELs (60,000–80,000 h in PEM water electrolyzers).

### 3.2 Mechanical Degradation

The mechanical degradation of an IEM is typically accompanied by another mechanism that results in the weakening of IEM mechanical properties, such as insufficient hydration causing the membrane to become dry and brittle or chemical degradation of the IEM polymer backbone as previously mentioned.^43^ Working with IEMs can be difficult because their fragile nature coupled with the need for frequent hydration necessitates a higher degree of caution and care; some researchers have suggested that difficulties associated with membrane handling play a significant factor in reduced cell performance.^44^ Additionally, mechanical degradation in the form of tears or pinholes can occur through hot pressing an MEA or overcompressing an MEA-based cell. In these scenarios, stray carbon fibers from GDEs can puncture the membrane. Taylor et al. demonstrated that this scenario can be effectively avoided by implementing a calendaring step on the GDE prior to electrode assembly, flattening out most or all the stray fibers.^45^ Cracks, tears, and pinholes can also occur during cell operation due to changes in temperature and humidity;^21^ while the fluctuations may be relatively easy to avoid in small-scale tests, they are likely to pose issues at larger scales where, without targeted intervention, temperature gradients will exist within both the cell and membrane. Upon the formation of pinholes or tears, the gas crossover rate is greatly increased, electrolyzer FE and EE drop significantly, and electrical shorting can occur, especially in the case of a larger tear.^41,45^

Interfacial delamination is a mechanical degradation mechanism that is primarily associated with BPMs. In general, differences in the mechanical properties of the AEL and CEL, such as swelling rates, can result in delamination.^21^ Forward-bias BPMs, where the AEL is in contact with the cathode GDE, suffer from the buildup of salts, CO_2_, formic acid, and H_2_O at the interface of the two exchange layers; delamination will occur if these are not removed from the cell at a quick rate.^34^ Hu et al. were able to mitigate this pervasive failure mode by employing a composite forward bias BPM with a PCEM. They utilized a 25 cm^2^ zero-gap cell, generating protons on the anode via the hydrogen oxidation reaction and formate ions on the cathode via the CO_2_R. The PCEM layer enabled formate ions to travel through the AEM, combine with protons at the BPM interface and interstitial CEM pores to form formic acid, and leave the cell via the anode GDE and flow field. This configuration achieved >75% FE to formic acid while operating at <2 V and 300 mA/cm^2^. A 55 h stability test at 200 mA/cm^2^ demonstrated the effectiveness of this delamination mitigation strategy.^34^

BPMs running in reverse bias (CEL contacting the cathode GDE) are at a much lower risk of delamination as water is dissociated into OH^–^ and H^+^ at the AEL|CEL interface, which then diffuse to their respective GDEs. Upon delamination, the electrochemical performance of an electrolyzer drops dramatically, resulting in rapid cell failure.

## 4 Durability Testing and Degradation Characterization

### 4.1 General Approach to Durability Testing and Characterizing Degradation

The wide variety of existing cell degradation mechanisms that can result in similar electrochemical responses adds further to the complexity of research targeted at improving the lifetime of CO_2_R devices. Therefore, it is paramount that a general approach for durability testing and degradation characterization be established to aid in this effort. In the following section, we propose one such approach that includes suggestions targeted at identifying IEM degradation mechanisms (Figure 4). A summary of our suggested characterizations can be found in Table 2, including some additional characterizations that may prove useful in certain testing scenarios. Furthermore, we bring forth the prospective utilization of operando methodologies in combination with accelerated testing procedures.

**Figure 4:**
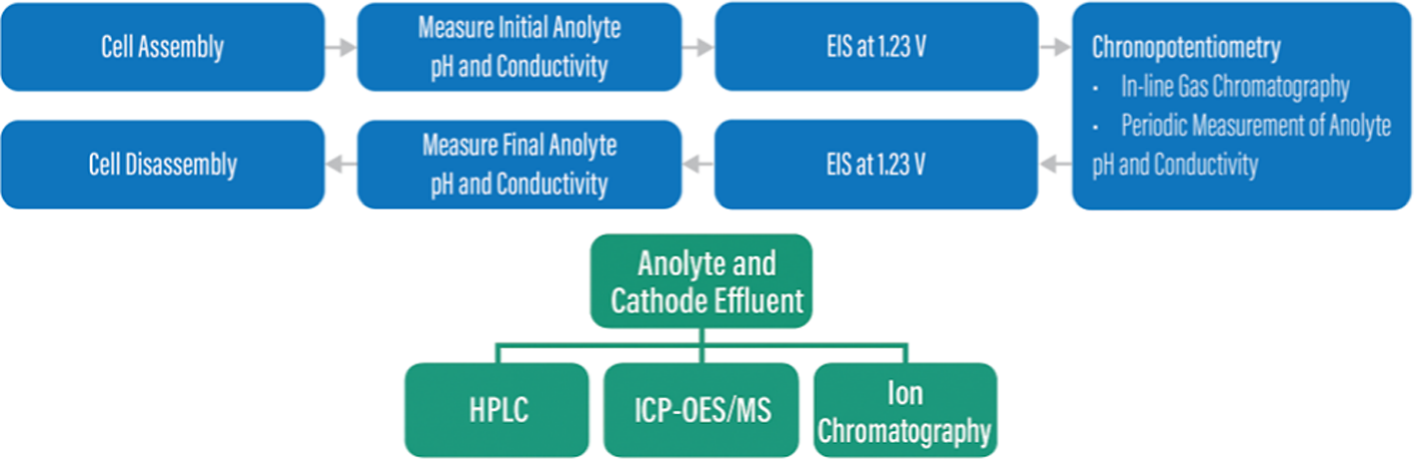
Diagram detailing a suggested characterization methodology to investigate the durability and/or degradation of a CO_2_ electrolyzer. Included are a recommended general procedure to follow during durability testing (blue) and additional characterizations that will help elucidate degradation mechanisms (green).

**Table 2:** Suggested Characterizations to Use When Performing CO_2_ Electrolysis Durability Tests to Investigate Degradation Mechanisms, along with the Related Advantages and Disadvantages of Utilizing Them^a^

characterizations	advantages	disadvantages
Essential Characterizations		
in-line GC	determine gaseous product selectivity throughout durability testing	GC columns are sensitive to CO_2_ and require bakeout periods between sets of samples
		water will cause damage, necessitating heat exchangers and/or water filters to be built within the CO_2_R system
HPLC	characterize liquid products	regular use becomes expensive
		instrument requires regular maintenance
anolyte conductivity and pH	provides information related to anolyte salt concentration	results can be misleading depending on the initial anolyte salts used. KOH will quickly convert to K_2_CO_3_ and KHCO_3_, convoluting the results
	monitors for changes in the reaction environment and kinetics	
	samples can be taken periodically throughout durability testing	
Frequently Useful Characterizations		
EIS at 1.23 V	nondestructive	limited information can be pulled from EIS due to the complexity of the system without inclusion of reference electrodes
	HFR provides insight into the electrolyte and IEM resistance	requires a potentiostat to implement
ICP-OES/MS	quantify ionomer/binder dissolution, catalyst leaching, contaminants, etc., from anolyte samples and cathode effluent	expensive and time-consuming to run
		ICP-MS will be required for trace amounts, requiring a long sample digestion
in-plane IEM conductivity	nondestructive	requires potentiostat
	must be performed before/after cell assembly/disassembly	may be misrepresentative of the more important through-plane conductivities
	provides potential insights into chemical degradation of charged functional groups in IEMs	sufficient catalyst adherence to the membrane during testing and/or a loss of membrane integrity can render post-mortem measurements impractical
Situationally Useful Characterizations		
Raman spectroscopy	can be used on IEM before and after durability testing to characterize polymer degradation	small scan size will require many sampling points and long characterization times
		low penetration depth
Pb underpotential deposition	provides catalyst surface area for silver	forms Cu–Pb alloys
	possibility of Pb contamination	can be performed on an assembled cell

a The characterizations have been categorized by their usefulness, being designated as essential, frequently useful, or situationally useful.

Electrochemical impedance spectroscopy (EIS) can be performed before and after cell assembly and durability testing to evaluate the in-plane conductivity of the IEM, assess changes in charge-transfer resistance, and detect signs of chemical degradation affecting the ionic functional groups.^38,46^ Measurement of the through-plane IEM conductivity can provide more relevant conductivity information than that of the in-plane counterpart, as anion transport primarily occurs through the membrane thickness. However, accurate through-plane measurements require a specialized cell, and post-mortem measurements become even more challenging when catalyst layers remain attached to one or both sides of the membrane after electrolysis, as this necessitates corrections for nonmembrane resistances.^30^ For these reasons, we generally recommend in-plane conductivity measurements before and after cell assembly since the exposed membrane surface is typically larger than the electrode area, enabling electrical contact in regions free of the adhered catalyst. Furthermore, EIS can be conducted at 1.23 V, the thermodynamic potential for water electrolysis (oxygen evolution reaction and HER), which is lower than the onset of significant Faradaic reactions in CO_2_ electrolysis systems at standard temperature and pressure (STP) conditions. The high-frequency intercept of the Nyquist plot represents the cell’s ohmic resistance (*R*_Ω_), which encompasses resistance from membrane conductivity, bulk electronic resistances from the flow fields and wiring, contact resistances at the interfaces between flow fields, GDEs, membrane interfaces, etc.^47^ EIS at this potential thus serves as a nondestructive diagnostic to establish a baseline resistance profile. Increases in *R*_Ω_ following testing may indicate membrane dehydration, swelling, or delamination, while a substantial decrease could suggest membrane failure through short-circuiting or electrolyte flooding.

CO_2_R cells can be operated under low, medium, and high current conditions to gain insight into any rate-dependent degradation mechanisms that may be present. In-line gas chromatograph measurements of gas products should be performed throughout durability tests, as changes in electrochemical performance may be accompanied by variations in product selectivity. In addition, it is recommended that electrolyte samples be taken periodically (∼1–2 times every 24 h) and measured for conductivity to gain insight into salt concentrations; a large loss of conductivity can indicate a high degree of salt precipitation in the system. Furthermore, cathode effluent and anolyte should be collected periodically when possible and examined with high-performance liquid chromatography, ion-exchange chromatography (IC), and/or inductively coupled plasma optical emission spectrometry (or ICP mass spectrometry (ICP-MS)) to quantify liquid products, binder dissolution, catalyst leaching, etc.^4^ The release of fluoride is a commonly utilized metric in PEM fuel cell and PEM water electrolyzer durability studies that is correlated to membrane and ionomer degradation.^46^ Moreover, IC can be used to detect other IEM degradation products such as sulfur, trimethylbenzyl ammonium (TMBA), dimethylamine (DMA), monomethylamine (MMA), and ammonium (NH_4+_).^48^

### 4.2 Future Incorporation of *Operando* Characterizations in Combination with ASTs/ADTs

The prospective utilization of *operando* characterizations on CO_2_R devices operating at industrially relevant currents is a promising avenue to accurately identify and address degradation mechanisms, resulting in the acceleration of increased device lifetimes. Promising real-time insights into catalyst structural evolutions, catalyst microenvironments, ion transport through membranes, and reaction intermediates/mechanisms, the use of *operando* characterizations with GDE-based cells will undoubtedly play a large role in reaching the durability goals of >35,000 h.^1,28^ Unfortunately, most *operando* studies to date have utilized H-cell configurations, while those using GDE-based cells displayed poor electrochemical performances, resulting from the cell modifications required for the techniques. Nevertheless, the increasing interest in the use of *operando* characterizations and the success demonstrated in the *operando* characterization of fuel cells and water electrolyzers suggest that the design of modified GDE-based CO_2_ reduction cells, capable of *operando* characterization at industrially relevant current densities, is a likely occurrence in the relatively near future. Hursan and Janaky recently published a detailed review of current state-of-the-art *operando* methods and how they could be utilized to address many of the largest challenges facing industrial application of CO_2_ reduction.^28^ Herein, we will briefly discuss one *operando* method that seems especially promising toward this end, neutron radiographic imaging (NRI).

NRI is a nondestructive imaging technique that has been previously utilized to characterize the water management within fuel cells, water electrolyzers, and, more recently, CO_2_ electrolyzers. In the latter application, NRI has been used to monitor membrane hydration levels, water distribution across an MEA, and the formation of gas bubbles within electrolyte layers.^28^ Furthermore, high-resolution NRI (HR-NRI) has been utilized in a zero-gap AEM-based electrolyzer to study water distribution and salt precipitation at the cathode, both of which can be considered vital to extending the longevity of CO_2_R devices. Unfortunately, NRI requires a neutron facility, making it much too expensive to perform over long durability tests, with some publications already achieving steady cell operation for over 166 days.^49^ To make the application of this powerful *operando* method financially feasible, we put forth the idea that NRI, among other similarly promising techniques (such as X-ray absorption spectroscopy), should be applied to the development of accelerated stress and durability testing (AST/ADT) for CO_2_ electrolysis.

ADTs and ASTs have been reported for other electrochemical technology fields such as fuel cells, water electrolysis, and chlor-alkali electrolysis, but very few have been reported for CO_2_R.^50^ As device lifetimes continue to increase, ADTs will become necessary to achieve industry-required durability in a timely manner.^1^ *Operando* characterization is a promising route to scrutinize the durability-limiting degradation mechanisms within GDE-based CO_2_ electrolyzers; furthermore, the identification of these mechanisms will prove beneficial to the development of ADTs/ASTs that will be required to financially enable the continued use of expensive *operando* methods, as device lifetimes reach scales upward of 10,000 h (>416 days). This synergetic relationship between accelerated test methods and *operando* characterizations will help catapult CO_2_R devices toward achieving both industrially relevant electrochemical performance and durability, enabling a more rapid integration of CO_2_ reduction technology into commercial use.

## 5 Summary

Currently, there are many industrial waste streams that contain high concentrations of CO_2_. Utilizing CO_2_ electrolysis in combination with renewable energy sources offers a promising route to convert this waste into fuels and chemicals, increasing industrial efficiency and providing an additional resource stream to enable the expansion of domestically made products. Due to advancements in cell configuration, electrocatalyst, and electrode technologies, industrially relevant electrochemical performance has been consistently achieved in the literature; however, current devices fall drastically short of the required lifetimes, with recent TEA modeling quoting a required durability of >4 years (>35,000 h).^1^ This perspective provides a brief overview of industrially relevant CO_2_ electrolyzer cell configurations, commonly incorporated IEMs, and degradation mechanisms. To encourage increased research efforts into the study of MEA-based CO_2_R degradation mechanisms, especially those relating to the vital IEM component, we suggest a general approach to characterizing degradation in the context of CO_2_ electrolyzer durability testing and degradation mechanism scrutiny. Furthermore, we encourage the future adoption of operando characterizations in combination with the development of ASTs/ADTs, postulating that the two methodologies will be of increased benefit when used in tandem.
